# Clinical characteristics and early identification of augmented renal clearance in PICU patients with severe sepsis associated with MRSA infection

**DOI:** 10.3389/fped.2024.1433417

**Published:** 2024-11-25

**Authors:** Haonan Wang, Jiaqing Li, Xian Li, Han Li, Yinglang He, Rui Tan, Xuejian Mei, Haoyu Zha, Mingxing Fan, Shuangshuang Peng, Nan Hou, Zhe Li, Yue Wang, Chen Ji, Yao Liu, Hongjun Miao

**Affiliations:** ^1^ Department of Emergency and Pediatric Intensive Care Unit, Children’s Hospital of Nanjing Medical University, Nanjing, Jiangsu, China; ^2^ Department of Anesthesiology, Children’s Hospital of Nanjing Medical University, Nanjing, Jiangsu, China; ^3^ Institution of Pediatrics, Nanjing Medical University, Nanjing, Jiangsu, China; ^4^ School of Public Health, Nanjing Medical University, Nanjing, Jiangsu, China; ^5^ Department of Pharmacy, Children’s Hospital of Nanjing Medical University, Nanjing, Jiangsu, China

**Keywords:** severe sepsis, MRSA, vancomycin, augmented renal clearance, prediction model

## Abstract

**Objectives:**

To investigate the epidemiological characteristics of Augmented Renal Clearance (ARC) in severe sepsis children with MRSA infection and find risk factors to establish a model predicting ARC onset in PICU.

**Design:**

Retrospective study, in which ARC was defined by estimated glomerular filtration rate (eGFR) measured by the modified Schwartz formula above 130 ml/min/1.73 m^2^. Univariable and multivariable logistic regression analyses were performed to find the predictor for ARC. Multi-strategy modeling was used to form an early prediction model for ARC, which was evaluated by the area under the ROC curve (AUC), accuracy (ACC) and other indicators.

**Setting:**

One China PICU.

**Patients:**

Severe sepsis children with MRSA infection admitted to PICU from May 2017 to June 2022 at Children's Hospital of Nanjing Medical University.

**Interventions:**

None.

**Measurements and main results:**

125 of 167 (74.9%) patients with severe sepsis with MRSA infection have occurred ARC during the hospitalization of PICU, of which 44% have an absolute decrease in vancomycin trough level (VTL), patients with ARC have a longer length of stay in both hospital and PICU, lower VTL and require longer anti-infective treatment. 20 different models were established for the early recognition of ARC. Among them, the best performer had an AUC of 0.746 and a high application prospect.

**Conclusion:**

ARC is a phenomenon significantly underestimated in pediatric patients with severe sepsis associated with MRSA infection, which can affect 74.9% of these patients and affects the process of anti-infection treatment and clinical outcomes. To achieve early prediction only by specific risk factors is unreliable, a model based on Multivariate Logistic Regression in this study was chosen to be used clinically.

## 1 Introduction

Severe sepsis and septic shock, as some most burdensome situations of infection and inflammation, had an increasing incidence in pediatrics in recent years and could cause 16%–25% of deaths (1, 2), remaining a challenging public health problem. Attributing to the global misuse of antibiotics, resistant bacteria have been a rapid emergency and pose a major obstacle to the curation of severe sepsis (3). Methicillin-resistant *Staphylococcus aureus* (MRSA) is a common multidrug-resistant organism (MDRO) in PICU and has the highest case-fatality rate among Gram-positive organisms (2). Vancomycin, a glycopeptide antibiotic, was still considered luckily effective in treating most severe infections caused by Gram-positive bacteria including MRSA, but the latest studies found that vancomycin in standard doses may fail to achieve the expected effects in 55%–69% of critically ill children (4).

This phenomenon is considered to be related to augmented renal clearance (ARC). ARC is common in critically ill adults or children, featured by increased creatinine clearance (CrCl) and elimination of drugs metabolized through the kidney (5). The incidence of ARC in PICU was reported to reach 7.8%–78% in different criteria and subgroups, and the modified Schwartz equation was used most and regarded as precise enough to document CrCl (6, 7). It's confirmed that ARC affects the pharmacokinetics of vancomycin and varieties of commonly used drugs in PICU, and population-based pharmacokinetic models were established to make medications more individual for those with ARC (8, 9).

The exact mechanism of ARC remained unknown, as options vary, hyperperfusion based on fluid resuscitation or vasopressor support was hypothesized to occupy an important position in the process.

However, few studies focused on ARC with children facing both severe sepsis and MRSA. Existing results on ARC risk factors were not representative of these patients (10, 11). As a unique but not rare subgroup, they relied more on antibiotic therapy, but it's unknown how ARC will perform under the co-effects, or say competition, of worse primary hemodynamic level and more active vasopressors and fluid therapy.

Therefore, to understand the characteristics of these patients, it is necessary to analyze the risk factors for ARC in severe sepsis with MRSA infection in PICU, and it is also urgent to establish a precise and reliable prediction model guiding to identify the high-risk ones developing therapeutic drug monitoring (TDM) and related treatment.

## 2 Methods

### 2.1 Study design

This was a retrospective cohort study. Severe sepsis children with MRSA infection admitted to PICU from July 2017 to May 2022 were collected, and they were all treated in the Department of Emergency and Intensive Care Medicine, Children's Hospital of Nanjing Medical University. This study was conducted following the Helsinki Declaration of 1975 and approved by the Medical Institutional Review Board of the Children's Hospital of Nanjing Medical University (approval No. 202008056-1, approval date: 2020-08-07) and informed consent was waived in this study.

Subjects were included if they: (1) aged from 28 days to 18 years; (2) met the criteria of severe sepsis by International Pediatric Sepsis Consensus Conference 2005 (12); (3) had the province of MRSA infection confirmed by etiological examination and antimicrobial susceptibility testing (AST); (4) had at least one TDM for vancomycin during the PICU hospitalization; and (5) had normal baseline renal function test data within 24 h admitted in PICU and at least another one during hospitalization.

Subjects were excluded if they: (1) were treated in PICU for less than 24 h (including in-hospital death, giving up salvage or being transferred out), (2) were performed an elective surgery before being transferred to PICU, (3) had congenital urinary malformation or inborn errors of metabolism (IEM) affecting kidney function (RFT), (4) had more than 30% data loss of continuous variables or any categorical variable in the statistics.

Finally, 167 cases were included in our study, as detailed in Supplementary File 1 along with the evidence sources of MRSA infection.

### 2.2 Data collection

Data collected in this study included demographic and anthropometric data, laboratory test data and medical intervention data. All demographic, anthropometric and laboratory tests (excluding RFT used to evaluate the highest in-PICU eGFR and VTL) were documented within the first 24 h admitted to PICU, unless otherwise specified. Vancomycin dose was recorded as total daily dose/kg. The estimated glomerular filtration rate (eGFR) was calculated by the Modified Schwartz Formula (13). PRSIM-III, PEWS and other commonly used assessments were recorded at the first PICU 24 h.

With the help of the Vancomycin Advanced AUC Calculator—GlobalRPH developed by Dr. Girgis's team, we estimated AUC_24_ based on VTL and Bayesian analysis. Vancomycin clearance (CL_vancomycin_) was estimated based on the work of Avedissian's team (7).

### 2.3 Definition

ARC was defined to have a maximum eGFR >130 mL/min/1.73 m^2^ during hospitalization. Patients were divided into the ARC group and the non-ARC group.

### 2.4 Statistical analysis

Variables were set for the class and disordered multinomial variables in modeling, and univariable Logistic regression was carried out for risk factors. The variance inflation factor (VIF) was used for the collinearity diagnosis, and VIF ≥10 was considered as serious collinearity. Multivariate Logistic regression was performed after highly collinear variables were eliminated. Indicators collected in the first 24 h of admission to PICU were used to establish an early warning model for ARC during hospitalization. The data set was randomly divided into the training set and the test set according to 6:4. The model was built in the training set and the internal parameter verification was carried out in the test set.

Multi-strategy Modeling was performed in our work, we used Univariable Logistic Regression, LASSO Regression, Random Forest (RF), Learning Vector Quantization (LVQ) and Forward Stepwise Regression (FSR) for feature screening, respectively. Multivariate Logistic regression, RF, XGBoost and support vector machine (SVM) were respectively used to establish the prediction model based on the above features. Classification accuracy was evaluated by area under the ROC curve (AUC). The parameters of each model were tested by accuracy (ACC), sensitivity (TPR), specialty (TNR) and false positive rate (FPR) calculating the mean square error in the confusion matrix.

Normal distribution continuous variables were represented by Mean ± SD, and non-normal distribution continuous variables were represented by Median (Quartile). Two-tailed Student *t*-tests or Mann-Whitney *U*-tests and Chi-square tests or Fisher exact tests were used respectively for continuous and categorical variables.

All analyses were performed in R 4.2.2[R Core Team (2022). R: A language and environment for statistical, computing. R Foundation for Statistical Computing, Vienna, Austria. URL https://www.R-project.org/], and α as the threshold for statistical tests was set at 0.05.

## 3 Results

### 3.1 Patient characteristics

Table 1 showed the baseline characteristics of the patients in this study, in which a total of 167 patients were included. 103 of 167 (61.7%) were males, and central nervous system diseases were the most common protopathy (44.3%, 74 of 167). The median length of stay (LOS) and median length of stay in PICU (LOS-ICU) were respectively 27 days [interquartile range (IQR), 18–42 days] and 15 days (IQR, 10–31 days). 19.8% (33 of 167) died of diseases in this hospitalization.

**Table 1:** The baseline characteristics in patients with or without augmented renal clearance.

Variable	All (*n* = 167)	Non-ARC (*n* = 42)	ARC (*n* = 125)	*P*-value
Gender = male (%)	103 (61.7)	26 (61.9)	77 (61.6)	1.000
Age [median (IQR)]	18.0 [4.0, 54.00]	4.50 [2.0, 17.3]	23.0 [5.0, 74.0]	*<0*.*001*
≤3 month	41 (24.6%)	17 (40.5%)	24 (58.5%)	
3 month–2 years	60 (35.9%)	17 (40.5%)	43 (71.7%)	
2 years–8 years	31 (18.6%)	4 (9.5%)	27 (87.1%)	
>8 years	35 (21.0%)	4 (9.5%)	31 (88.6%)	
Height (cm, median [IQR])	81.0 [60.0, 111.0]	63.0 [53.8, 78.0]	88.0 [65.0, 117.0]	*<0*.*001*
Weight (kg, median [IQR])	10.5 [6.0, 16.0]	6.9 [4.6, 10.4]	11.5 [7.3, 21.0]	*<0*.*001*
BMI [kg/m^2^, median (IQR)]	16.17 [14.30 18.26]	16.16 [14.42, 18.62]	16.17 [14.17, 18.08]	0.669
BSA [m^2^, median (IQR)]	0.95 [0.65, 1.37]	0.69 [0.56, 0.91]	1.05 [0.72, 1.48]	*<0*.*001*
Protopathy (%)				0.111
Respiratory	57 (34.1)	19 (45.2)	38 (30.4)	
Central nervous system	74 (44.3)	14 (33.3)	60 (48.0)	
Cardiovascular	5 (3.0)	3 (7.1)	2 (1.6)	
Digestive	7 (4.2)	1 (2.4)	6 (4.8)	
Hemopathic	11 (6.6)	1 (2.4)	10 (8.0)	
Others	13 (7.8)	4 (9.5)	9 (7.2)	
Disease death (%)	33 (19.8)	11 (26.2)	22 (17.6)	0.324
LOS [day, median (IQR)]	27.0 [18.0, 42.0]	19.0 [13.5, 27.8]	31.0 [20.0, 51.0]	*<0*.*001*
LOS-ICU [day, median (IQR)]	15.0 [10.0, 31.0]	13.0 [11.0, 19.3]	18.0 [10.0, 34.0]	*0*.*049*
Hospitalization cost [¥, median (IQR)]	76,186.0 [49,627.5, 134,173.0]	65,040.5 [46,048.3, 93,072.3]	82,203.0 [52,275.0, 156,979.0]	*0*.*023*

ARC, augmented renal clearance; IQR, interquartile range; BMI, body mass index; BSA, body surface area; LOS, length of stay (in hospital); LOS-ICU, length of stay in PICU.

### 3.2 Augmented renal clearance

125 of 167 (74.9%) had ARC during the hospitalization. Gender and protopathic were not significantly different between the groups, while patients in the ARC group were older than the non (23.0 months [5.0 vs.74.0] vs. 4.5 months [2.0, 17.3], *p* < 0.001) and so do other age-associated indicators shown in Table 1.

### 3.3 Medical assessments and intervention

As shown in Table 2, children in the ARC group had higher PCIS scores, lower SOFA scores, lower PELOD-2 scores and lower P-MODS scores. There was no significant difference in PRISM III, PEWS, SIRS scores between the two groups (*P* ≥ 0.05).

**Table 2:** Medical assessments and intervention.

Medical intervene	All (*n* = 167)	Non-ARC (*n* = 42)	ARC (*n* = 125)	*P*-value
PRISM III [median (IQR)]	13.0 [10.0, 17.0]	12.50 [10.0, 17.8]	13.0 [10.0, 16.0]	0.951
PCIS [median (IQR)]	88.0 [86.0, 92.0]	87.00 [84.0, 91.5]	90.0 [86.0, 94.0]	*0*.*012*
PEWS [median (IQR)]	5.0 [4.0, 6.0]	5.00 [4.0, 6.0]	5.0 [4.0, 6.0]	0.279
pSOFA [median (IQR)]	5.0 [3.0, 6.0]	5.00 [3.3, 8.8]	4.0 [3.0, 6.0]	*0*.*026*
qSOFA [mean (SD)]	1.65 (0.78)	1.86 (0.84)	1.58 (0.74)	*0*.*042*
PELOD-2 [mean (SD)]	3.26 (3.47)	4.26 (3.86)	2.93 (3.27)	*0*.*030*
P-MODS [mean (SD)]	3.78 (2.41)	4.83 (2.88)	3.43 (2.13)	*0*.*001*
SIRS [median (IQR)]	11.0 [8.0, 12.0]	11.0 [10.0, 12.0]	10.0 [8.0, 12.0]	0.282
APACHE II (median [IQR])	24.0 [19.5, 27.0]	25.0 [23.0, 28.8]	23.0 [19.0, 26.0]	*0*.*045*
Use of vasoactive drugs in the first PICU 24 h (%)	39 (23.4)	17 (40.5)	22 (17.6)	*0*.*005*
Mechanical ventilation (%)	58 (34.7)	19 (45.2)	39 (31.2)	0.143
PaO2/FiO2 [mmHg, median (IQR)]	225.0 [181.7, 345.0]	223.9 [155.3, 258.3]	225.0 [189.7, 350.0]	0.067
Vancomycin dose [mg/kg/day, median (IQR)]	40.0 [39.2, 42.2]	40.00 [40.0, 44.0]	40.00 [39.2, 41.7]	0.357
Vancomycin course [day, median (IQR)]	11.0 [7.0, 19.0]	9.0 [7.0, 12.8]	12.0 [8.0, 20.0]	*0*.*027*
Vancomycin trough level [VTL, μg/mL, median (IQR)]	7.2 [4.7, 11.8]	10.3 [7.2, 20.3]	6.2 [4.3, 10.2]	*<0*.*001*
VTL less than 5 μg/mL (%)	50 (29.9)	6 (14.3)	44 (35.2)	*0*.*018*
VTL less than 10 μg/mL (%)	114 (68.3)	21 (50.0)	93 (74.4)	*0*.*006*
AUC_24_/MIC	273.2[204.8, 375.7]	353.0 [255.1, 569.9]	249.6 [199.6, 329.3]	*<0*.*001*
CL_vancomycin_	2.19[1.18 3.87]	1.06 [0.63, 1.59]	2.96 [1.52, 4.74]	*<0*.*001*

AUC_24_, 24-h area under the concentration-time curve; based on Bayesian Analysis supported by Vancomycin Advanced AUC Calculator - GlobalRPH developed by Dr. Girgis's team; MIC, minimum inhibitory concentration, assumed as 1 mg/L in empiric therapy; CL_vancomycin_, vancomycin clearance (L/h).

Thirty-nine of 167 (23.4%) received vasoactive drugs in the first PICU 24 h, and fifty-eight of 167 (34.7%) received mechanical ventilation(MV) in the first PICU 24 h. Median daily dose of vancomycin in ARC-group and non-ARC group weren't significantly different (*p* = 0.357), while the VTL of the ARC group was lower than non-ARC group [6.2 μg/mL (IQR, 4.3, 10.2) vs. 10.3 μg/mL (IQR, 7.2, 20.3), *p* < 0.001], and patients in the ARC group were more likely to have subtherapeutic exposure (35.2% vs. 14.3% in VTL <5 μg/mL, *p* = 0.018; 74.4% vs. 50.0 in VTL <10 μg/mL, *p* = 0.006). Especially, the analysis for vancomycin only focused on the first course if the patient received several independent vancomycin therapy during the study dates, median vancomycin course of the ARC group was longer than non-ARC group [12.0 days (IQR, 8.0–20.0) in the ARC group vs. 9.0 days (IQR, 7.0–12.8) in the non-ARC group, *p* = 0.027].

For more details of TDM, the AUC_24_/MIC of vancomycin in the ARC group is significantly lower than in the non-ARC group [249.6 (199.6, 329.3) vs. 353.0 (255.1, 569.9), *P* < 0.001], typically below 400. With the support of the vancomycin clearance PK formula from the multicenter study led by Dr. Sean N Avedissian'team, the estimated CL_vancomycin_ in the ARC group is significantly higher than that in the non-ARC group [2.96 (1.52, 4.74) vs. 1.06 (0.63, 1.59), L/h, *P* < 0.001].

### 3.4 Anthropometric and laboratory test data

The two groups’ anthropometric and laboratory test data were all shown in Supplementary File 2.

### 3.5 Risk factors of ARC

**Figure 1:**
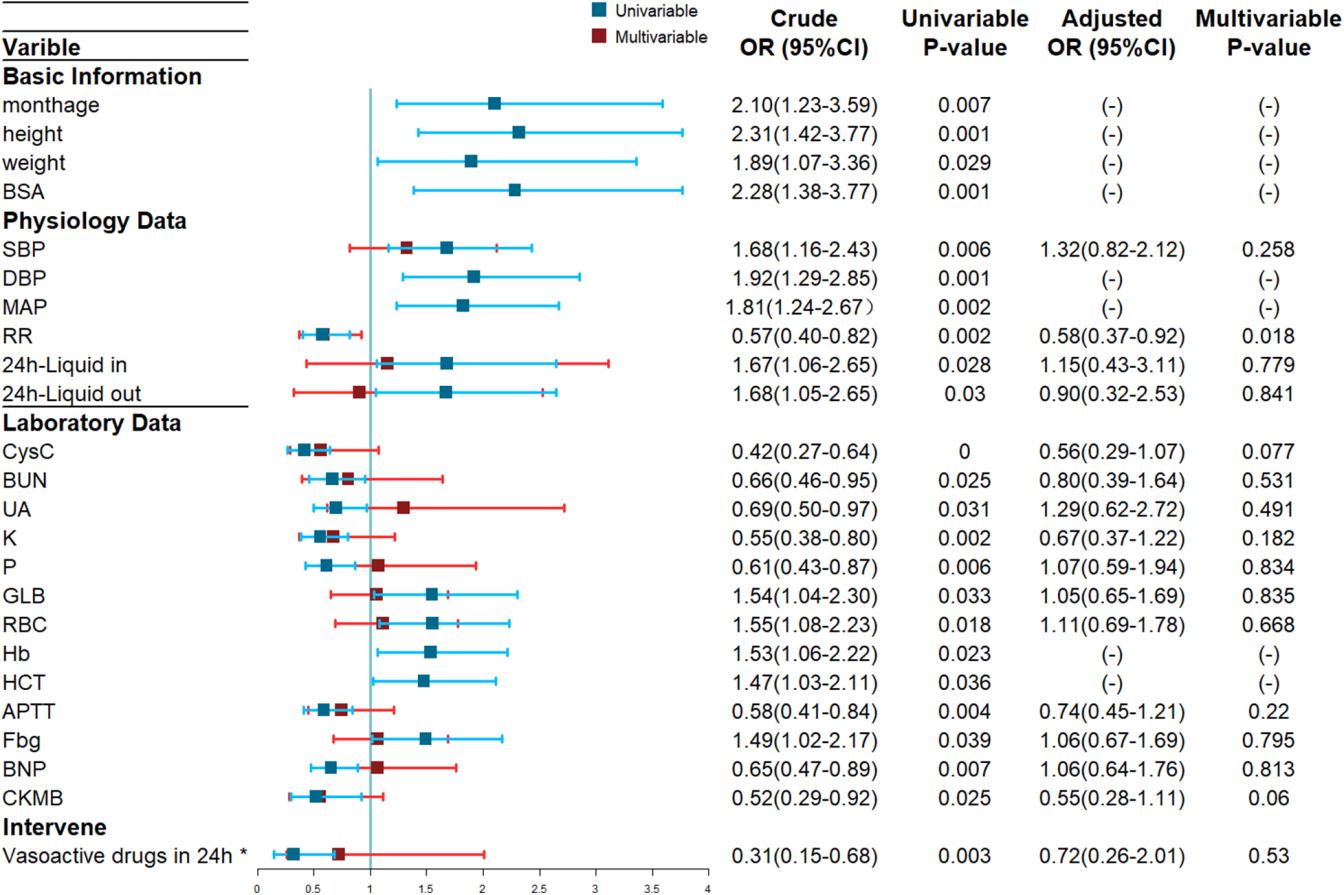
Forest Plot of ARC Risk Factors based on Univariate and Multivariate Logistic Regression Analyses. Blue lines: crude odds ratios (OR) from the univariate logistic regression. Red lines: adjusted OR from the multivariate logistic regression, values not shown due to multicollinearity confirmed by VIF testing. *Vasoactive drugs includes epinephrine, norepinephrine, dopamine, and dobutamine et al.

As shown in Figure 1, 24 potential risk factors by univariate logistic regression analysis were found and then performed collinearity diagnosis. Age (VIF = 12.4), height (VIF = 27,293.7), weight (VIF = 1,187.1), BSA (VIF = 38,472.3), DBP (VIF = 12.8), MAP (VIF = 29.3), Hb (VIF = 21.7) and HCT (VIF = 23.6) were excluded and finally 16 indexes were included. Multivariate Logistic regression analysis showed respiratory rate as the only independent predictor (OR = 0.58, 95%CI: 0.37–0.92, *p* = 0.018).

### 3.6 Early prediction model of ARC

Table 3 showed different combinations of variables included in the prediction model based on different feature screening methods. Among the 20 models established noted in Table 4, there were three models with AUC above 0.7 of which the highest was 0.75, seven models with accuracy above 75% of which the highest was 81%, two models with sensitivity above 75% of which the highest was 85% and fifteen models with specificity above 75% of which the highest was 96%.

**Table 3:** Feature selection for prediction models.

Feature selection method	Number of features	Features
Univariable logistic regression	16	SBP, RR, 24 h-liquid-in, 24 h-liquid-out, CysC, BUN, UA, K, P, GLB, RBC, APTT, Fbg, BNP, CKMB, Vasoactive drugs
LASSO & logistic regression	4	Height, Cr, K, Vasoactive drugs
Random forest	10	Weight, age, 24 h-liquid-in, RBC, height, BSA, Cr, MAP, P, IBIL
Importance based feature selectin based on LVQ	10	Height, BSA, age, weight, K, MAP, DBP, CysC, SBP, 24 h-urine volume
Forward stepwise regression	12	Height, TT, TC, RBP, BUNCR, TBIL, MV, GLB, MCHC, HR, ALP, gender

All features associated data were collected in the first 24 h admitted in PICU.

**Table 4:** Early prediction model for ARC in PICU patients with severe bacterial infections.

	Classifier	Feature selection	Number of features	ACC	AUC	FPR	TPR	TNR
1	Random forest	OOB	23	0.73 (0.61–0.83)	0.54 (0.43–0.66)	0.80	0.20	0.88
2	Random forest	RF-10	10	0.70 (0.58–0.81)	0.55 (0.42–0.67)	0.73	0.27	0.83
3	Random forest	RF-5	5	0.64 (0.55–0.76)	0.48 (0.37–0.60)	0.80	0.20	0.77
4	Random forest	LASSO + Logistic	4	0.79 (0.67–0.88)	0.70 (0.56–0.84)	0.47	0.53	0.87
5	Logistic	LASSO + Logistic	4	0.79	0.75 (0.60–0.89)	0.15	0.85	0.60
6	Logistic	RF-10	10	0.73	0.71 (0.55–0.86)	0.23	0.77	0.60
7	Logistic	RF-5	5	0.54	0.59 (0.43–0.76)	0.54	0.46	0.80
8	Logistic	FSR-All	12	0.52	0.62 (0.46–0.79)	0.58	0.42	0.87
9	Logistic	FSR-5	5	0.66	0.67 (0.49–0.84)	0.29	0.71	0.47
10	Logistic	IFS-LVQ10	10	0.66	0.62 (0.45–0.79)	0.37	0.63	0.73
11	Logistic	IFS-LVQ5	5	0.64	0.59 (0.42–0.76)	0.35	0.65	0.60
12	SVM	LASSO + Logistic	4	0.78	0.62 (0.49–0.75)	0.67	0.33	0.90
13	SVM	Univariable Logistic Regression	16	0.79	0.58 (0.47–0.69)	0.80	0.20	0.96
14	SVM	FSR	12	0.76	0.59 (0.46–0.71)	0.73	0.27	0.90
15	XGBoost	None	ALL	0.79 (0.67–0.88)	0.63 (0.50–0.76)	0.67	0.33	0.92
16	XGBoost	Univariable Logistic Regression	16	0.72 (0.59–0.82)	0.56 (0.43–0.68)	0.73	0.27	0.85
17	XGBoost	LASSO + Logistic	4	0.81 (0.69–0.89)	0.73 (0.60–0.90)	0.40	0.60	0.87
18	XGBoost	IFS-LVQ10	10	0.69 (0.56–0.79)	0.54 (0.41–0.67)	0.73	0.27	0.81
19	XGBoost	RF	10	0.73 (0.61–0.83)	0.59 (0.46–0.72)	0.67	0.33	0.85
20	XGBoost	FSR	12	0.75 (0.63–0.84)	0.60 (0.47–0.73)	0.67	0.33	0.87

ACC, accuracy; AUC, area under the curve; FPR, false positive rate; TPR, true positive rate; TNR, true negative rate; OOB, out-of-bag; RF, random forest; FSR, forward stepwise regression; SVM, support vector machine.

**Figure 2:**
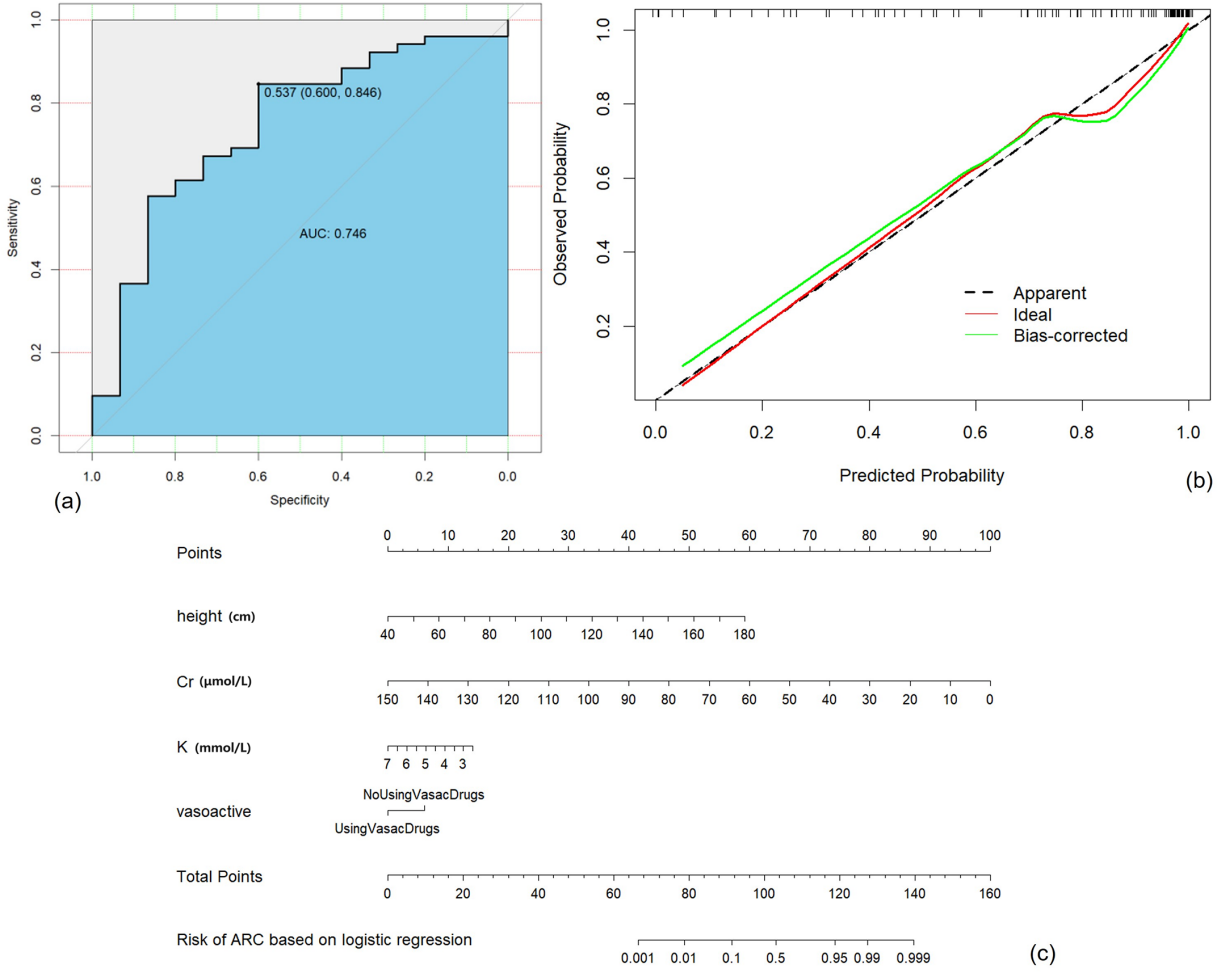
Construction, validation, and visualization of the clinical prediction model for augmented renal clearance (ARC) (Model 5). **(a)** ROC curve of the model. **(b)** Calibration curve of the model. **(c)** Nomogram of the model.

The Multivariable Logistic Regression Model with continuous variables screened by LASSO Regression and discrete variables screened by Univariable Logistic Regression (Model 5) showed the best AUC (0.75, 95% CI: 0.60–0.89), satisfying accuracy (ACC = 0.79), sensitivity (TPR = 84.6%) and specificity (TNR = 60.0%). The goodness of fit based on the Hosmer-Lemeshow test showed χ^2^ = 45 (*p* > 0.5), and the C index of the model was 0.90 (*p* < 0.001). The calibration curve showed that the observed results were in good agreement with the predicted results, details were showed in Figure 2.

## 4 Discussion

### 4.1 Incidence of ARC in children with severe sepsis with MRSA infection in PICU

In our study, 74.9% of the severe sepsis with MRSA infection had occurred ARC in PICU, of which the incidence was close to the highest interval reported now (14, 15). We believed that such a high prevalence is caused by the special physiological background of children and the pathological state caused by severe infection.

Firstly, the modified Schwartz Formula has higher accuracy than other equations in children and showed high consistency with the mGFR (16, 17). Most studies believed that this calculation would result in an underestimation of 19.8% (18), while very few prompted the opposite. The high incidence in this study is more unlikely due to the evaluation method. Secondly, it is reported that a higher incidence occurred in younger adults (19, 20), and some studies suggest that children were more likely to suffer from ARC than adults in diseases of similar severity (6). Thirdly, as a severe medical condition, many of the severe sepsis patients were treated with vasopressors and cardiotonic agents with the probability instead of the province of septic shock or sepsis-associated organ dysfunction, which may be potentially involved in the process of ARC.

### 4.2 Clinical impacts

In this study, the negative clinical effects of ARC can be divided into the following aspects: (1) Reduced blood concentration of vancomycin or other drugs and extended duration of drug use; (2) Extended length of stay in PICU and significantly increased medical expenses.

VTL in the ARC group was much lower than that in the non-ARC group, and the duration of medication was significantly extended when there was no significant difference in the daily dose and drug combination. VTL <5 μg/mL had nearly no antibacterial effect on almost any form of infection, it was observed that 35.2% and 74.4% in the ARC group showed VTL lower than 5 μg/mL and 10 μg/mL, which were significantly higher than those without ARC (14.3% and 50.0%). ARC leads to increased renal clearance of vancomycin at standard doses, which poses a possibility for anti-infective therapy failure. For additional TDM details, our non-invasive methods demonstrated that children in the ARC group had higher vancomycin clearance and lower AUC_24_/MIC. These findings provide more direct evidence of pharmacokinetic target failure, highlighting the challenges in achieving therapeutic goals in this population. In a worse-case scenario, subtherapeutic exposure may lead to a high risk of selective resistance (21, 22).

This is not an isolated phenomenon. The latest study showed that 55% of children with sepsis who were empirically treated with vancomycin had insufficient VTL, and higher eGFR was independently associated with subtherapeutic VTL (4). Therapeutic exposure to meropenem was not achieved in 80% of PICU sepsis patients with ARC (23). Among adult patients treated with piperacillin, only 58% achieved the basic pharmacodynamic target. In multivariate analysis, CrCl remains an important predictor of subtherapeutic concentration (24). It is noteworthy that vancomycin is one of the few drugs that can perform normalized TDM in PICU, and it is a good signpost. While it is not economical and convenient for other drugs to guide medication dose adjustment through TDM, they are all theoretically more or less affected by ARC and have been partially confirmed.

At present, most studies believe that the occurrence of ARC does not affect the survival outcome of patients (25, 26). In this study, there was still no statistical difference between the mortality rate of children with ARC and those without. However, evaluations including P-MODS, PELOD-2, PCIS and SOFA scores were significantly different between the groups, and the ARC group was predicted with a lower risk of death, less severe organ dysfunction and a more optimistic prognosis. Still, the length of stay and cost of hospitalization showed adversely. In conclusion, although ARC does not significantly and directly determine the survival outcome, it can extend the treatment process of patients, resulting in greater health risks and economic burden.

### 4.3 Risk factors for ARC in children with severe PICU infection

Univariable logistic regression suggested 24 potential risk factors and there were 16 left screened by VIF, and multivariate logistic regression only suggested respiratory rate as statistically significant which seemed to conflict with clinical experience, so it is unreliable to predict ARC in PICU patients with severe infections only by specific risk factors.

Potential risk factors, as shown in Table 4, could be summarized as age-related (e.g., Age, height, weight, BSA et al.), circulation-related (e.g., SBP, DBP, MAP, liquid intake and output volume, use of vasoactive drugs, BNP), renal function related (e.g., CysC, BUN, SCr, UA, K, P) and others. Among these factors, higher blood pressure, higher BNP and larger urine output represent healthy circulation and normal or upregulated renal perfusion, while lower serum creatinine and CysC partly indicate hyperfiltration of the glomeruli. Lower potassium and phosphate levels represent increased excretion due to higher urine output or tubular secretion function and partially reflect a more stable internal environment. Besides, older age in pediatrics represents more mature kidneys and better baseline renal function. It may also be due to older children having better general conditions when facing similarly severe illnesses. Nevertheless, in studies on ARC, age demonstrates contrasting trends between pediatric and adult patient groups, which is highly intriguing.

Notably, the concentration of CRP and PCT were not meaningful risk factors in this study, partly because patients in our cohort all burned in severe infection and inflammations so the effect of the inflammatory response is less obvious, if existing.

The study by Jason (27), Nei (28) and Baptista (10) suggested that lower age was an independent risk factor for ARC in severely infected adults. Age in our study was also an important factor, however, the older, the higher the risk of ARC, which is different from almost all studies based on adults. It could be explained by the development of renal function and better circulation in older children. As for many other significant factors like RBC, APTT, BNP and respiratory rate, it is difficult to explain whether they are accident differences of physiological conditions or undiscovered mechanisms. Undeniably, there were conclusions conflicted with our study like BMI in the study of He (29), young, male and high APACHE II scores in the study of Johnston (11),and other research, which may be due to specific patient groups. These hypotheses need to be further confirmed.

### 4.4 ARC's early prediction model

At present, in the modeling work of ARC, Rhoney (6) established pharmacokinetic models of commonly used drugs in ARC, but conducting TDM for each drug is not feasible. Li (30) established an eGFR prediction model with excellent performance applicable to the local population, but it required high real-time data and could not accurately predict the whole hospitalization events. Sean (7) did not limit the type of disease and successfully predicted the blood concentration of vancomycin in ARC patients, but could not predict the occurrence of ARC and any other drugs’ concentration. Nei (28) successfully established an early ARC prediction model for adult ICU with an excellent area under the curve (AUC = 0.95), but no similar studies have been conducted in pediatrics.

This study successfully established various ARC early warning models based on data within 24 h of admission into PICU. Finally, Model 5 was selected based on the best AUC, exceptional Accuracy and TPR performance. Our ARC prediction model incorporates four parameters: height, serum potassium, serum creatinine and the administration of vasoactive drugs. Height is considered closely associated with the renal size and baseline renal function of pediatric patients. Creatinine predominantly reflects glomerular filtration function, negatively correlated with the probability of occurrence of ARC. Serum potassium is an indicator for assessing electrolyte imbalances and partly predicting overall outcomes, while also partially reflecting renal tubular function. The use of vasoactive drugs signifies medical interventions influencing the upregulation of renal perfusion. All four factors included were available.

The early ARC prediction model established in this study can guide clinical judgment conveniently, quickly and intuitively. PICU physicians can assess the risk of ARC in severe sepsis patients with MRSA based on the model proposed in this study. They can enhance the monitoring of renal clearance rates and concentrations of certain specific medications appropriately to achieve a better prognosis.

## 5 Conclusion

ARC is a phenomenon significantly underestimated in pediatric patients with severe sepsis associated with MRSA infection, which can affect 74.9% of these patients. There were 16 potential risk factors screened by univariable logistic regression and collinearity diagnosis. Multiple-strategy modeling was performed to build prediction models for ARC in our study, and the final model based on LASSO and Logistic Regression includes four features (height, serum potassium, serum creatinine, the administration of vasoactive drugs) and demonstrates stable performance (AUC = 0.75, 95% CI: 0.60–0.89, ACC = 0.79, TPR = 84.6%, TNR = 60.0%). The model shows great potential for clinical application and can assess the risk of ARC occurrence during hospitalization using indicators within 24 h of pediatric patients entering the PICU.

## 6 Limitations

The cohort in our study was a serious and unique subgroup with a significantly high occurrence of ARC. The statistical results were credible and explainable, but more cases are required for the validation and optimization of the prediction model. Additionally, although our study is pioneering in using data in the 1st 24 h in PICU to predict the overall risk of ARC during hospitalization, there are still unanswered questions regarding when ARC occurs, how long it takes, and its severity. Further work is needed.

In addition, some systemic issues have not been addressed. Measured GFR or CrCl based on 24-h urine is usually unobtainable in the PICU, especially difficult to monitor dynamically. Moreover, almost all existing eGFR estimation methods are derived from patients with renal dysfunction, raising questions about their suitability for assessing renal function in ARC patients. Furthermore, it appears that we should assess the muscle mass *Z* score of each patient to avoid error use of the modified Schwartz formula, but this is also challenging in clinical practice, especially in PICU.

Compared to vancomycin, more drugs are not routinely monitored or technically challenging to monitor through TDM, so population pharmacokinetics models and clinical trials about dose optimization are anticipated. Until this significant endeavor is achieved, early identification by PICU physicians and the professional contributions of clinical pharmacists will greatly benefit patients. Therefore, the PICU team strongly requires the involvement of clinical pharmacists in ward rounds and medication management.

We included important TDM parameters such as VTLs, CL_vancomycin_ and AUC_24_/MIC to support some of our conclusions. However, we must acknowledge that estimating cannot replace actual measurements or pharmacokinetic analyses conducted by professionals. Unknown factors may work especially in such a unique patient group. Additionally, please note that there is still ongoing debate regarding the optimal AUC/MIC target in pediatric patients with specific subgroups, and which TDM metric is the most appropriate. As we mentioned earlier, clinical pharmacists are vital for the PICU team.

## Data Availability

The raw data supporting the conclusions of this article will be made available by the authors, without undue reservation.

## References

[1] Weiss SL Fitzgerald JC Pappachan J Wheeler D Jaramillo-Bustamante JC Salloo A Global epidemiology of pediatric severe sepsis: the sepsis prevalence, outcomes, and therapies study. Am J Respir Crit Care Med. (2015) 191(10):1147–57. 10.1164/rccm.201412-2323OC 25734408 PMC4451622

[2] Sehgal M Ladd HJ Totapally B. Trends in epidemiology and microbiology of severe sepsis and septic shock in children. Hosp Pediatr. (2020) 10(12):1021–30. 10.1542/hpeds.2020-0174 33208389

[3] Woolhouse M Waugh C Perry MR Nair H. Global disease burden due to antibiotic resistance—state of the evidence. J Glob Health. (2016) 6(1):010306. 10.7189/jogh.06.010306 27350872 PMC4920009

[4] Scully PT Lam WM Coronado Munoz AJ Modem VM. Augmented renal clearance of vancomycin in suspected sepsis: single-center, retrospective pediatric cohort. Pediatr Crit Care Med. (2022) 23(6):444–52. 10.1097/PCC.0000000000002918 35190502

[5] Udy AA Roberts JA Boots RJ Paterson DL Lipman J. Augmented Renal Clearance Implications for Antibacterial Dosing in the Critically Ill. Available online at: https://link.springer.com/article/10.2165/11318140-000000000-00000 (accessed July 14, 2022) 10.2165/11318140-000000000-00000 20000886

[6] Rhoney DH Metzger SA Nelson NR. Scoping review of augmented renal clearance in critically ill pediatric patients. Pharmacotherapy. (2021) 41(10):851–63. 10.1002/phar.2617 34431121

[7] Avedissian SN Bradley E Zhang D Bradley JS Nazer LH Tran TM Augmented renal clearance using population-based pharmacokinetic modeling in critically ill pediatric patients∗. Pediatr Crit Care Med. (2017) 18(9):e388–94. 10.1097/PCC.0000000000001228 28640009

[8] Nicolau DP De Waele J Kuti JL Caro L Larson KB Yu B Pharmacokinetics and pharmacodynamics of ceftolozane/tazobactam in critically ill patients with augmented renal clearance. Int J Antimicrob Agents. (2021) 57(4):106299. 10.1016/j.ijantimicag.2021.106299 33567333

[9] Abdel El Naeem HEM Abdelhamid MHE Atteya DAM. impact of augmented renal clearance on enoxaparin therapy in critically ill patients. Egypt J Anaesth. (2017) 33(1):113–7. 10.1016/j.egja.2016.11.001

[10] Baptista JP Martins PJ Marques M Pimentel JM. Prevalence and risk factors for augmented renal clearance in a population of critically ill patients. J Intensive Care Med. (2020) 35(10):1044–52. 10.1177/0885066618809688 30373438

[11] Johnston BW Perry D Habgood M Joshi M Krige A. Augmented renal clearance: a retrospective, cohort study of urinary creatinine clearance in critically ill patients in the United Kingdom. J Int Med Res. (2021) 49(5):3000605211015573. 10.1177/03000605211015573 34038207 PMC8161888

[12] Goldstein B Giroir B Randolph A. International pediatric sepsis consensus conference: definitions for sepsis and organ dysfunction in pediatrics*. Pediatr Crit Care Med. (2005) 6(1):2–8. 10.1097/01.PCC.0000149131.72248.E6 15636651

[13] Schwartz GJ Muñoz A Schneider MF Mak RH Kaskel F Warady BA New equations to estimate GFR in children with CKD. J Am Soc Nephrol. (2009) 20(3):629–37. 10.1681/ASN.2008030287 19158356 PMC2653687

[14] Barletta JF Mangram AJ Byrne M Hollingworth AK Sucher JF Ali-Osman FR The importance of empiric antibiotic dosing in critically ill trauma patients. J Trauma Acute Care Surg. (2016) 81(6):1115–21. 10.1097/TA.0000000000001211 27533906

[15] Udy AA Baptista JP Lim NL Joynt GM Jarrett P Wockner L Augmented renal clearance in the ICU. Crit Care Med. (2014) 42(3):520–7. 10.1097/CCM.0000000000000029 24201175

[16] Mian AN Schwartz GJ. Measurement and estimation of glomerular filtration rate in children. Adv Chronic Kidney Dis. (2017) 24(6):348–56. 10.1053/j.ackd.2017.09.011 29229165 PMC6198668

[17] de Souza V Cochat P Rabilloud M Selistre L Wagner M Hadj-Aissa A Accuracy of different equations in estimating GFR in pediatric kidney transplant recipients. Clin J Am Soc Nephrol. (2015) 10(3):463–70. 10.2215/CJN.06300614 25617430 PMC4348684

[18] Bhowmick R Rameshkumar R Ponnusamy M Rajaraman V Chidambaram M Sheriff A Modified Schwartz formula and 99m tc-DTPA plasma clearance methods to calculate glomerular filtration rate in critically ill children. Pediatr Nephrol. (2022) 37(4):899–906. 10.1007/s00467-021-05197-3 34546418

[19] Kawano Y Maruyama J Hokama R Koie M Nagashima R Hoshino K Outcomes in patients with infections and augmented renal clearance: a multicenter retrospective study. PLoS One. (2018) 13(12):e0208742. 10.1371/journal.pone.0208742 30532142 PMC6287846

[20] Chu Y Luo Y Jiang M Zhou B. Application of vancomycin in patients with augmented renal clearance. Eur J Hosp Pharm. (2020) 27(5):276–9. 10.1136/ejhpharm-2018-001781 32839259 PMC7447238

[21] Roberts JA Kruger P Paterson DL Lipman J. Antibiotic resistance—what’s dosing got to do with it? Crit Care Med. (2008) 36(8):2433–40. 10.1097/CCM.0b013e318180fe62 18596628

[22] You Only Find what you Look for The Importance of High Creatinine Clearance in the Critically Ill. Available online at: https://journals.sagepub.com/doi/10.1177/0310057×0903700123?url_ver=Z39.88-2003&rfr_id=ori:rid:crossref.org&rfr_dat=cr_pub%20%200pubmed (accessed July 14, 2022) 10.1177/0310057X0903700123 19157339

[23] Avedissian SN Skochko SM Le J Hingtgen S Harvey H Capparelli EV Use of simulation strategies to predict subtherapeutic meropenem exposure caused by augmented renal clearance in critically ill pediatric patients with sepsis. J Pediatr Pharmacol Ther. (2020) 25(5):413–22. 10.5863/1551-6776-25.5.413 32641911 PMC7337137

[24] Udy AA Varghese JM Altukroni M Briscoe S McWhinney BC Ungerer JP Subtherapeutic initial *β*-lactam concentrations in select critically ill patients. Chest. (2012) 142(1):30–9. 10.1378/chest.11-1671 22194591

[25] Udy AA Dulhunty JM Roberts JA Davis JS Webb SAR Bellomo R Association between augmented renal clearance and clinical outcomes in patients receiving β-lactam antibiotic therapy by continuous or intermittent infusion: a nested cohort study of the BLING-II randomised, placebo-controlled, clinical trial. Int J Antimicrob Agents. (2017) 49(5):624–30. 10.1016/j.ijantimicag.2016.12.022 28286115

[26] Huttner A Von Dach E Renzoni A Huttner BD Affaticati M Pagani L Augmented renal clearance, low β-lactam concentrations and clinical outcomes in the critically ill: an observational prospective cohort study. Int J Antimicrob Agents. (2015) 45(4):385–92. 10.1016/j.ijantimicag.2014.12.017 25656151

[27] Burnham JP Micek ST Kollef MH. Augmented renal clearance is not a risk factor for mortality in Enterobacteriaceae bloodstream infections treated with appropriate empiric antimicrobials. PLoS One. (2017) 12(7):e0180247. 10.1371/journal.pone.0180247 28678812 PMC5497982

[28] Nei AM Kashani KB Dierkhising R Barreto EF. Predictors of augmented renal clearance in a heterogeneous ICU population as defined by creatinine and cystatin C. Nephron. (2020) 144(7):313–20. 10.1159/000507255 32428906 PMC7371523

[29] He CY Qin YR Liu CJ Ren J Fan JS. Effect of augmented renal clearance on plasma concentration of vancomycin and treatment outcome in children with methicillin-resistant Staphylococcus aureus infection. Chin J Contemp Pediatr. (2019) 21(9):904–9. 10.7499/j.issn.1008-8830.2019.09.012 PMC7390248 31506151

[30] Li N Huang H Qian HZ Liu P Lu H Liu X. Improving accuracy of estimating glomerular filtration rate using artificial neural network: model development and validation. J Transl Med. (2020) 18(1):120. 10.1186/s12967-020-02287-y 32156297 PMC7063770

