# Supplementary material for: Clinical characteristics and early identification of augmented renal clearance in PICU patients with severe sepsis associated with MRSA infection
Source: Front Pediatr. 2024 Nov 25;12:1433417. doi: 10.3389/fped.2024.1433417 (PMC11629473; doi:10.3389/fped.2024.1433417)
Supplement: Supplementary file 1 [file Table1.pdf]

Supplementary file 2：Anthropometric and Laboratory Test Data

	All (n=167)	Without ARC (n=42)	With ARC (n=125)	P value
**GCS** (median [IQR])	13.00 [9.00, 13.00]	13.00 [9.00, 13.00]	13.00 [9.00, 13.00]	0.74
**T** (°C, median [IQR])	38.00 [37.00, 39.00]	37.45 [36.80, 38.75]	38.20 [37.10, 39.00]	0.096
**HR** (/min, median [IQR])	148.00 [127.50, 167.50]	153.00 [132.75, 177.25]	145.00 [125.00, 166.00]	0.089
**RR** (/min, median [IQR])	32.00 [26.00, 41.00]	36.00 [30.00, 47.75]	30.00 [26.00, 39.00]	***0.004***
**SBP** (mmHg, median [IQR])	105.00 [90.00, 115.50]	91.00 [85.25, 111.75]	106.00 [93.00, 117.00]	***0.007***
**DBP** (mmHg, mean (SD))	63.31 (16.45)	56.02 (16.24)	65.76 (15.85)	***0.001***
**MAP** (mmHg, median [IQR])	77.33 [64.16, 87.67]	65.67 [55.83, 80.25]	80.00 [67.00, 87.67]	***0.001***
**pH** (median [IQR])	7.40 [7.36, 7.43]	7.39 [7.34, 7.40]	7.40 [7.36, 7.45]	***0.006***
**Liquid in** (mL, median [IQR])	1017.00 [746.00, 1496.75]	865.50 [583.95, 1172.68]	1046.80 [820.40, 1599.30]	***0.007***
**Liquid out** (mL, median [IQR])	905.00 [522.00, 1332.50]	593.00 [375.00, 986.25]	920.00 [613.00, 1439.00]	***0.004***
**Urine Volume** (mL, median [IQR])	755.00 [452.50, 1134.00]	496.50 [340.50, 888.75]	830.00 [540.00, 1219.00]	***0.003***
**Net Input** (mL, median [IQR])	125.00 [-18.00, 293.70]	115.80 [-26.75, 301.00]	143.00 [-16.00, 292.00]	0.897
**$PaCO_2$**(mmHg, median [IQR])	35.40 [26.85, 40.00]	39.40 [30.57, 40.83]	34.80 [26.40, 40.00]	0.066
**PaO_2** (mmHg, median [IQR])	90.00 [77.25, 138.50]	90.00 [69.60, 116.75]	91.20 [78.50, 141.00]	0.117
**SpO_2** (%, median [IQR])	99.00 [95.15, 100.00]	98.75 [95.00, 100.00]	99.00 [96.00, 100.00]	0.529
**Lac** (mmol/L, median [IQR])	0.90 [0.50, 1.90]	0.85 [0.50, 2.88]	0.90 [0.50, 1.80]	0.554
**CRP** (mg/L, median [IQR])	32.05 [8.00, 115.50]	41.50 [8.00, 119.25]	31.00 [8.00, 113.00]	0.559
**PCT** (procalcitonin, ng/mL, median [IQR])	1.58 [0.19, 10.48]	2.99 [0.16, 20.01]	1.30 [0.26, 8.77]	0.359
**WBC** (10^9/L, median [IQR])	11.47 [6.40, 19.02]	9.96 [8.29, 19.12]	11.65 [6.27, 18.95]	0.891
**LN%** (median [IQR])	24.40 [13.20, 41.15]	32.90 [11.23, 46.45]	23.70 [14.30, 39.10]	0.545
**MO%** (median [IQR])	6.70 [5.25, 9.85]	6.30 [4.75, 9.88]	6.90 [5.30, 9.80]	0.849
**NE%** (median [IQR])	65.80 [48.15, 78.45]	59.50 [41.42, 80.72]	67.70 [49.70, 77.20]	0.561
**EO%** (median [IQR])	0.10 [0.00, 0.80]	0.30 [0.10, 0.65]	0.10 [0.00, 0.80]	0.268
**BA%** (median [IQR])	0.10 [0.10, 0.25]	0.10 [0.10, 0.30]	0.10 [0.10, 0.20]	0.51
**LN#** (10^9/L, median [IQR])	2.28 [1.29, 4.47]	2.78 [1.33, 6.13]	2.05 [1.29, 4.11]	0.453
**MO#** (10^9/L, median [IQR])	0.79 [0.33, 1.40]	0.84 [0.33, 1.64]	0.78 [0.33, 1.36]	0.748
**NE#** (10^9/L, median [IQR])	7.33 [3.24, 12.39]	5.78 [3.47, 12.51]	7.65 [3.24, 12.38]	0.839
**EO#** (10^9/L, median [IQR])	0.02 [0.00, 0.08]	0.02 [0.01, 0.11]	0.02 [0.00, 0.07]	0.36
**BA#** (10^9/L, median [IQR])	0.01 [0.01, 0.03]	0.01 [0.01, 0.04]	0.02 [0.01, 0.03]	0.715
**RBC** (10^{12}/L, mean (SD))	3.74 (0.76)	3.50 (0.85)	3.83 (0.72)	***0.016***
**Hb** (g/L, mean (SD))	103.17 (20.42)	96.86 (19.61)	105.29 (20.33)	***0.02***
**HCT** (%, mean (SD))	31.82 (5.96)	30.12 (5.64)	32.38 (5.98)	***0.033***
**MCV** (fL, median [IQR])	85.10 [80.15, 91.15]	85.85 [80.45, 94.85]	85.00 [79.70, 90.30]	0.22
**MCH** (pg, mean (SD))	27.78 (2.98)	28.17 (3.44)	27.65 (2.82)	0.332
**MCHC** (g/L, mean (SD))	323.95 (15.18)	320.93 (16.13)	324.96 (14.78)	0.137
**RDW** (fL, median [IQR])	43.40 [39.75, 48.15]	45.05 [39.80, 49.65]	43.20 [39.80, 47.20]	0.217
**RDWCV** (%, median [IQR])	14.40 [13.40, 15.60]	14.80 [13.74, 15.78]	14.10 [13.20, 15.50]	0.177
**PLT** (10^9/L, median [IQR])	243.00 [125.50, 410.00]	338.00 [166.25, 508.25]	217.00 [117.00, 356.00]	***0.021***
**PCT** (Platelet hematocrit, %, median [IQR])	0.26 [0.15, 0.42]	0.32 [0.17, 0.51]	0.25 [0.15, 0.38]	0.189
**MPV** (fL, median [IQR])	10.30 [9.70, 10.95]	10.35 [9.62, 11.12]	10.20 [9.70, 10.90]	0.99
**PDW** (fL, median [IQR])	11.50 [10.20, 13.60]	11.60 [10.12, 13.17]	11.40 [10.40, 13.70]	0.829
**PLCR** (%, median [IQR])	26.60 [20.95, 32.80]	26.05 [21.00, 31.18]	26.70 [21.10, 33.00]	0.87
**ALT** (median [IQR])	25.00 [14.00, 62.50]	26.00 [15.25, 61.25]	25.00 [13.00, 63.00]	0.937
**AST** (median [IQR])	43.00 [25.50, 78.50]	46.50 [28.25, 83.82]	42.00 [25.00, 75.00]	0.463
**ALP** (median [IQR])	137.00 [95.50, 200.50]	140.50 [114.00, 223.50]	134.00 [93.00, 194.00]	0.159
**GGT** (median [IQR])	29.00 [13.50, 80.00]	41.00 [19.25, 87.50]	25.00 [13.00, 70.00]	0.199
**Che** (median [IQR])	4974.00 [3616.50, 6349.00]	4923.50 [3728.25, 6154.50]	5007.00 [3536.00, 6558.00]	0.844
**LDH** (median [IQR])	378.00 [274.00, 552.00]	421.50 [281.75, 597.00]	366.00 [274.00, 516.00]	0.159
**CK** (median [IQR])	66.00 [33.00, 160.00]	96.00 [39.00, 176.50]	63.00 [31.00, 159.00]	0.231
**CKMB** (median [IQR])	26.00 [18.00, 38.50]	33.00 [22.25, 74.50]	24.00 [17.00, 36.00]	***0.007***

**HBDH** (median [IQR])	272.00 [201.00, 420.50]	295.00 [214.25, 475.00]	266.00 [198.00, 381.00]	0.303
**TP** (mean (SD))	58.29 (9.93)	56.85 (8.33)	58.78 (10.40)	0.277
**ALB** (mean (SD))	35.34 (6.96)	35.99 (6.07)	35.12 (7.25)	0.483
**GLB** (median [IQR])	21.50 [17.95, 26.95]	20.45 [17.35, 24.12]	22.10 [18.30, 27.90]	0.062
**A/G** (median [IQR])	1.62 [1.23, 2.04]	1.71 [1.40, 2.14]	1.55 [1.15, 2.03]	0.057
**Glu** (median [IQR])	5.21 [4.31, 6.67]	4.69 [4.00, 5.54]	5.59 [4.47, 6.75]	0.05
**TBIL** (median [IQR])	6.40 [4.22, 11.49]	7.85 [5.05, 13.08]	6.30 [4.00, 11.00]	0.338
**DBIL** (median [IQR])	3.10 [2.08, 5.60]	3.50 [2.63, 6.30]	2.90 [1.92, 5.10]	0.212
**IBIL** (median [IQR])	3.11 [1.80, 5.80]	3.13 [1.85, 7.05]	3.00 [1.80, 5.44]	0.419
**PA** (median [IQR])	0.10 [0.06, 0.16]	0.10 [0.06, 0.14]	0.11 [0.05, 0.16]	0.741
**BUN** (median [IQR])	3.50 [2.70, 4.95]	3.90 [2.88, 6.25]	3.44 [2.70, 4.80]	0.142
**UA** (median [IQR])	199.00 [137.50, 284.50]	211.50 [139.00, 338.25]	197.00 [137.00, 260.00]	0.171
**Cr** (median [IQR])	25.00 [18.15, 38.05]	30.50 [23.00, 50.97]	23.00 [17.00, 35.00]	***0.001***
**BUN/Cr** (median [IQR])	33.36 [26.90, 46.72]	32.94 [24.69, 45.71]	34.52 [27.23, 47.85]	0.403
**CysC** (median [IQR])	1.03 [0.84, 1.30]	1.27 [1.06, 1.52]	0.94 [0.81, 1.20]	***<0.001***
**RBP** (median [IQR])	16.36 [9.40, 26.25]	16.16 [10.55, 22.55]	16.36 [9.07, 26.45]	0.916
**K** (median [IQR])	4.21 [3.64, 4.80]	4.61 [3.99, 5.35]	4.11 [3.59, 4.68]	***0.003***
**Na** (median [IQR])	135.60 [132.50, 138.00]	134.45 [131.45, 137.75]	136.00 [133.00, 138.00]	0.281
**Cl** (median [IQR])	98.60 [95.05, 102.20]	96.65 [93.20, 101.33]	98.90 [96.50, 102.60]	***0.032***
**Ca** (median [IQR])	2.24 [2.07, 2.37]	2.26 [2.07, 2.42]	2.24 [2.07, 2.34]	0.4
**Mg** (median [IQR])	0.91 [0.84, 1.00]	0.92 [0.85, 1.04]	0.91 [0.83, 1.00]	0.508
**P** (median [IQR])	1.25 [0.99, 1.68]	1.58 [1.18, 1.81]	1.18 [0.97, 1.50]	***0.004***
**TG** (median [IQR])	1.22 [0.84, 1.75]	1.10 [0.77, 1.67]	1.31 [0.87, 1.77]	0.112
**TC** (median [IQR])	2.99 [2.27, 3.68]	2.64 [2.09, 3.68]	3.00 [2.30, 3.67]	0.326
**HDL** (median [IQR])	0.76 [0.51, 1.09]	0.86 [0.58, 1.15]	0.73 [0.50, 1.07]	0.119
**PT** (median [IQR])	13.00 [11.60, 15.30]	12.80 [11.62, 15.23]	13.10 [11.60, 15.30]	0.681
**INR** (median [IQR])	1.15 [1.04, 1.33]	1.15 [1.04, 1.34]	1.16 [1.04, 1.33]	0.684
**APTT** (median [IQR])	36.30 [30.95, 42.85]	40.70 [34.02, 46.48]	34.80 [30.80, 41.10]	***0.005***
**Fbg** (median [IQR])	3.17 [2.14, 4.51]	2.90 [1.93, 3.90]	3.37 [2.21, 4.57]	0.054
**TT** (median [IQR])	15.00 [13.45, 17.00]	15.30 [14.20, 16.77]	14.70 [13.20, 17.00]	0.141
**cTnI** (median [IQR])	0.01 [0.01, 0.03]	0.02 [0.01, 0.06]	0.01 [0.01, 0.03]	***0.008***
**Myo** (median [IQR])	18.40 [7.70, 66.85]	20.10 [9.10, 76.80]	16.80 [7.60, 66.70]	0.649
**CKMB mass** (median [IQR])	2.70 [0.95, 9.15]	3.35 [1.60, 17.78]	2.10 [0.80, 6.80]	***0.025***
**BNP** (median [IQR])	137.00 [47.00, 475.00]	221.50 [78.75, 1190.00]	97.00 [40.00, 323.00]	***0.005***
