# Supplementary material for: Clinical characteristics and early identification of augmented renal clearance in PICU patients with severe sepsis associated with MRSA infection
Source: Front Pediatr. 2024 Nov 25;12:1433417. doi: 10.3389/fped.2024.1433417 (PMC11629473; doi:10.3389/fped.2024.1433417)
Supplement: Supplementary file 2 [file Image1.pdf]

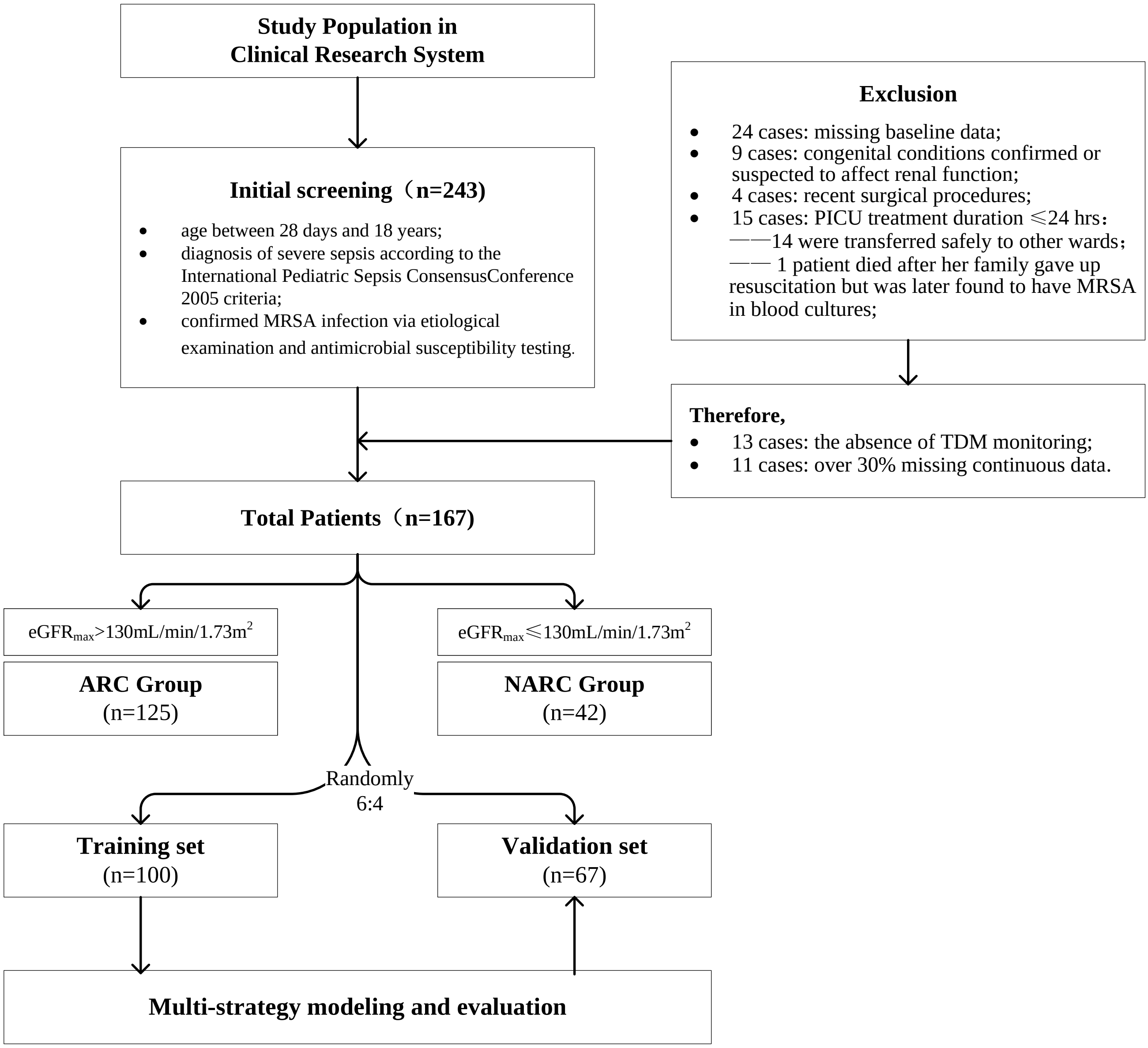

<u>Evidence Sources of MRSA Infection:</u>

We found MRSA in sputum or BALF in 70.1% (117) of patients, 29.3% (49) in blood, 3.0% (5) in urine, 4.2% (7) in CSF, 0.01% (1) in blood catheter, and 0.05% (8) in other specimens, including abscess drainage, wound secretions, and bone marrow.

<u>Details of Inclusion & Exclusion:</u>

A total of **243 cases** met the inclusion criteria. We excluded：**24 cases** due to missing baseline LFT and RFT data (resulting from laboratory tests being conducted outside the specified study timeframe for transferred patients, incomplete data transfer due to independent laboratory information systems, or, in a few cases, the absence of relevant orders); **9 cases** were excluded because of congenital conditions that could or were suspected to affect renal function (1 of TINU syndrome, 1 of Alport syndrome, and 7 cases with other confirmed pathogenic gene abnormalities that may cause multi-organ dysfunction or secondary damage to renal function);

4 patients were excluded due to recent surgical procedures; **15 cases** were excluded because of too short in-PICU treatment duration (14 were transferred safely to specialized wards, and 1 patient died after her family gave up resuscitation but was later found to have MRSA in blood cultures).

Therefore, **13 cases** were excluded due to the absence of TDM monitoring; **11 cases** were excluded due to over 30% missing continuous variable data, as detected by the statistical analysis software.

Finally, **167 cases** were included in our study, as detailed in our manuscript, they were divided into the **ARC group (n=125)** and **Non-ARC (n=42) group** based on whether the hightest eGFR was greater than 130ml/(min·1.73m^2) during PICU treatment. Then total patients were randomly divided into Training set (n=100) and Validation set (n=67) for subsequent analysis.
